# Virtual Reality for Enhancing Self-Compassion and Reducing Burnout Among Mental Health Care Professionals: Protocol for a Pilot Feasibility Randomized Controlled Trial

**DOI:** 10.2196/105754

**Published:** 2026-10-19

**Authors:** Rawan Iriqat, Andre Faro, Stephanie Tarle, Shavon Moore, Wael Al-Delaimy

**Affiliations:** 1 Herbert Wertheim School of Public Health and Human Longevity Science University of California San Diego La Jolla, CA United States; 2 Department of Psychology Universidade Federal de Sergipe Aracaju, Sergipe Brazil; 3 Department of Psychiatry University of California, San Diego La Jolla, CA United States

**Keywords:** virtual reality, burnout, self-compassion, implementation science, digital mental health

## Abstract

**Background:**

Mental health care professionals working in high-demand clinical environments are frequently exposed to patient suffering and trauma, placing them at increased risk for burnout and secondary traumatic stress. Compassion-focused cognitive behavioral therapy (CF-CBT) has shown promise in reducing psychological distress and enhancing self-compassion; however, traditional delivery methods may be limited by time, accessibility, and resource constraints. Virtual reality (VR) may provide an immersive and accessible approach for delivering compassion-focused interventions.

**Objective:**

The primary objective is to evaluate the feasibility and acceptability of a self-administered VR-enhanced CF-CBT (VR-CF-CBT) intervention for mental health care professionals. Secondary exploratory objectives are to estimate changes in self-compassion, burnout, and secondary traumatic stress (STS), as well as between-group differences that can inform the design of a future definitive trial.

**Methods:**

A 2-arm randomized controlled pilot trial will be conducted among mental health care professionals. Participants will be randomized to either a VR-CF-CBT intervention or an active comparison condition consisting of self-guided CF-CBT delivered via digital booklets. Participants will complete 8 brief sessions (approximately 10 minutes each) over a 4- to 6-week period. Exploratory clinical outcomes will include changes in self-compassion, burnout, and STS measured using validated self-report instruments. Primary feasibility and acceptability outcomes will include recruitment, retention, intervention adherence, session completion, participant satisfaction, and scores on the Acceptability of Intervention Measure, Intervention Appropriateness Measure, and Feasibility of Intervention Measure.

**Results:**

The study was funded in April 2026. As of October 2026, 38 participants had provided consent and enrolled. Recruitment for a second wave of participants is ongoing, and data collection is in progress. Enrollment and data collection are expected to continue through 2026, with data analysis and dissemination of findings anticipated by spring 2027.

**Conclusions:**

This pilot study will provide information on the feasibility and acceptability of delivering CF-CBT through immersive VR to mental health care professionals. Findings will help identify whether modifications are needed before a larger trial and provide preliminary estimates to inform the design of future efficacy testing.

**Trial Registration:**

ClinicalTrials.gov NCT07666867; https://clinicaltrials.gov/study/NCT07666867

**International Registered Report Identifier (IRRID):**

DERR1-10.2196/105754

## Introduction

Health care professionals frequently work in environments characterized by high levels of stress and repeated exposure to patient suffering, placing them at increased risk for burnout, emotional exhaustion, and secondary traumatic stress (STS) [1]. These risks may be especially high among mental health professionals and clinicians working in high-demand clinical settings, where repeated exposure to trauma and emotional distress can accumulate over time [2,3]. Chronic exposure to these stressors has been associated with difficulties in emotional regulation, increased cognitive rigidity, compassion fatigue, and reduced occupational well-being [4].

Beyond its impact on individual well-being, burnout also affects the health care system. Mental health care professionals experiencing burnout are more likely to disengage from their work, report reduced productivity, and consider leaving their positions, which may contribute to workforce shortages and reduced health care capacity [5]. These challenges are particularly important given that nearly 40% of the US population lives in a Mental Health Professional Shortage Area [6].

Several interventions have been developed to address burnout and work-related stress among health care professionals, including mindfulness training and psychoeducation [7,8]. While these approaches can be beneficial, they often rely heavily on sustained attention, verbal processing, and abstract visualization skills, which may be more difficult to engage during periods of chronic stress or emotional exhaustion.

Virtual reality (VR) has emerged as a promising tool for delivering psychological interventions through immersive and interactive experiences [9,10]. Emerging evidence suggests that VR-based interventions may support emotional regulation, mindfulness, resilience, and compassion while reducing stress and psychological distress [11-15]. These characteristics suggest that VR may be a useful modality for delivering compassion-focused interventions to health care professionals experiencing burnout and STS.

The present study is grounded in cognitive behavioral theory (CBT), which proposes that emotional and behavioral responses are shaped not only by external events but also by how individuals interpret and respond to those experiences [16-18]. Within health care settings, repeated exposure to patient suffering may contribute to maladaptive cognitive and emotional patterns, including excessive self-criticism, heightened threat perception, emotional withdrawal, and rigid thinking [19]. These processes have been linked to burnout, STS, and diminished psychological well-being among health care professionals.

Conceptually, this study is informed by a framework in which repeated exposure to patient suffering and trauma contributes to STS, including symptoms such as emotional distress, intrusive thoughts, and hyperarousal [20,21]. Over time, these experiences may contribute to emotional exhaustion, burnout, and reduced occupational functioning. As stress accumulates, health care professionals may experience a lower capacity for empathy and self-compassion, further affecting both personal well-being and quality of care. Interventions that strengthen self-compassion may help interrupt this process by promoting adaptive emotional regulation and resilience in the face of ongoing occupational stress.

Empathy and self-compassion are therefore conceptualized as important psychological processes that support sustainable and emotionally engaged care. Empathy allows health care professionals to remain responsive to patients while maintaining appropriate emotional boundaries, whereas self-compassion promotes adaptive responses to personal distress and may reduce excessive self-criticism and emotional exhaustion [22-24]. Previous research suggests that these skills can be strengthened through structured cognitive and behavioral interventions, including compassion-focused approaches that address self-criticism and shame [25-27].

Compassion-focused cognitive behavioral therapy (CF-CBT) is designed to target these mechanisms by strengthening self-compassion and improving emotional regulation. Drawing on principles from CF-CBT, brief immersive VR sessions may enhance engagement, support experiential learning, and provide a psychologically safe environment in which participants can practice compassion-focused skills while observing and modifying their cognitive and emotional responses in real time [8,9,28,29].

CBT-based approaches may be particularly well suited for adaptation to VR, because their core components are structured and operationalized to brief interventions focused on specific psychological processes [17,30]. Accordingly, this study will evaluate the feasibility and acceptability of a structured but flexible, self-guided VR-enhanced CF-CBT (VR-CF-CBT) intervention and explore preliminary changes in self-compassion, burnout, and STS among health care professionals working in high-demand clinical environments.

## Methods

### Study Design

This is a pilot randomized controlled trial that evaluates the feasibility and acceptability, and explores the preliminary clinical effects, of a VR-enhanced CF-CBT-based intervention for mental health care professionals experiencing work-related stress and burnout. Study participants are randomly assigned in a 1:1 ratio to either the intervention group, which receives VR experiences targeting cognitive distortions based on CF-CBT principles, or the control group, which receives the same CF-CBT content through self-guided digital booklets.

The intervention consists of 8 self-guided brief sessions (approximately 10 minutes) completed over approximately 4 to 6 weeks, and no specialized interventionists are required for intervention delivery. Participants in both groups receive the same core therapeutic content, with the primary difference being the mode of delivery. Study findings will be reported in accordance with the CONSORT (Consolidated Standards of Reporting Trials) guidelines [31]. This protocol is reported in accordance with the SPIRIT (Standard Protocol Items: Recommendations for Interventional Trials) guidelines [32] (Multimedia Appendix 1).

### Participants, Settings, and Timeline

Participants are recruited from University of California San Diego (UCSD) Health and affiliated health care settings; recruitment efforts may be expanded to other community clinics in San Diego if necessary. Eligible participants include mental health care professionals, including psychiatrists, psychologists, therapists or counselors; social workers; and other clinicians providing mental health care, who have practiced clinical care for at least 1 year and reside in San Diego to facilitate VR headset distribution.

Individuals are excluded if they have a medical condition that would prevent safe use of VR technology (eg, a history of severe motion sickness, uncontrolled seizure disorders, or other contraindications to VR use), do not provide direct patient care in their practice, or are currently participating in another intervention study targeting similar outcomes.

Potential participants are identified through study advertisements, institutional listserves, departmental communications, and referrals from collaborating health care organizations. Interested individuals complete an eligibility screening questionnaire and are provided with study information and an electronic informed consent form prior to enrollment.

### Sample Size

We aim to recruit approximately 52 mental health care professionals, with about 26 participants per group. This target was selected during study planning after a review of prior VR intervention literature and is consistent with the sample sizes used in early-stage VR studies [33]. As this is a pilot feasibility trial, the planned sample will be used primarily to evaluate feasibility and acceptability and to provide preliminary estimates of outcome variability and other parameters needed to inform the design of a future definitive trial [34]. The study is not designed or powered to provide definitive evidence of efficacy.

### Randomization and Blinding

Participants are randomized in a 1:1 ratio to either the VR-CF-CBT intervention group or the digital booklet–based CF-CBT control group using the Sealed Envelope Simple+ randomization service with variable block sizes of 4 and 6 [35]. No stratification is used. Randomization occurs after participants provide informed consent and complete baseline assessments.

Because of the nature of the intervention, participants and study personnel administering the intervention cannot be blinded to group assignment. To reduce the potential for bias, outcome assessments will be collected electronically using standardized self-report measures. Data analyses will be conducted using deidentified study data. Where feasible, investigators responsible for statistical analyses will remain blinded to group allocation until the planned analyses have been completed.

### Intervention

The intervention consists of a self-administered VR-CF-CBT program designed to reduce burnout and STS and to target cognitive distortions among mental health care professionals. VR-CF-CBT integrates principles of CBT, compassion-focused therapy, and mindfulness principles within immersive virtual environments.

The intervention will be delivered using Meta Quest 3S headsets. VR content was developed in collaboration with a VR development company and members of the study team with expertise in CBT, compassion-based interventions, digital mental health, and frontline psychiatric and behavioral health practice. The VR scenarios were designed to reflect challenging situations that mental health care professionals may encounter in their daily work. Practicing mental health professionals contributed to the development and refinement of the scenarios to help ensure that they reflected realistic clinical experiences and workplace stressors. Prior to implementation, the VR program underwent testing to assess usability, comprehension, and acceptability. Patients and members of the public were not involved in the design, conduct, reporting, or dissemination of this study.

The duration of the intervention is informed by established CBT frameworks, which commonly involve approximately 8 to 12 sessions and allow sufficient opportunity for skill acquisition and repeated practice while maintaining feasibility for participants with demanding schedules [16,17]. Prior VR-CBT studies have also used multiple-session intervention formats [36,37].

The duration of individual sessions was informed by a systematic review indicating that VR self-compassion interventions often include brief immersive experiences, with beneficial effects observed following sessions of approximately 10 minutes or less [33]. On the basis of this evidence, the present study uses short VR sessions lasting approximately 10 minutes delivered across 8 sessions over 4 to 6 weeks.

Participants assigned to the VR condition will have individual access to a headset throughout the intervention period and may complete sessions at times that fit their schedules within the 4- to 6-week intervention window. To maintain a standardized therapeutic progression, VR scenarios will be completed sequentially, with each module becoming available only after the preceding module has been completed.

Sessions follow a consistent 4-part structure: an introduction, an immersive scenario, guided reflection, and a debrief. They begin with a brief overview of the cognitive pattern or skill being addressed and its connection to stress, empathy, self-compassion, and burnout. Participants then engage in an immersive scenario that mirrors challenges commonly encountered in health care settings. Self-reflection triggers are integrated throughout the experience to help participants recognize automatic thoughts, emotional responses, and behavioral reactions as they occur. Each session concludes with a guided debrief that helps participants connect their experiences in VR to real-world clinical practice while reinforcing self-compassion, emotional regulation, and empathic engagement.

VR-CF-CBT focuses on 5 cognitive distortions that are commonly linked to burnout and STS: mind reading (2 sessions), personalization (2 sessions), emotional reasoning (1 session), catastrophizing (2 sessions), and fortune telling (1 session). Throughout the intervention, participants learn to recognize these thinking patterns and practice responding to them in more balanced and compassionate ways. Mindfulness techniques are integrated across all sessions to help participants develop greater awareness of their thoughts and emotions, increase tolerance for uncertainty, and respond more flexibly to emotionally challenging situations.

### Control

Participants randomized to the control group will receive the same core CF-CBT content through self-guided digital booklets rather than immersive VR. The booklet-based intervention mirrors the cognitive targets, educational material, reflective exercises, and mindfulness content included in the VR scenarios. Delivering equivalent therapeutic content across groups allows the study to evaluate the added contribution of immersive VR technology beyond traditional self-guided CF-CBT.

### Harms and Safety Measures

Adverse events will be assessed throughout the study through participant self-report and direct communication with the research team. Potential harms include simulator sickness, dizziness, emotional discomfort, or distress associated with intervention content. All adverse events will be documented and reviewed by the study team.

To minimize motion-related discomfort, VR exposure will be introduced gradually. Participants will be asked to familiarize themselves with the VR headset, and the Virtual Reality Sickness Questionnaire (VRSQ) will be administered after the first VR use to monitor tolerability and guide continued participation [38]. Participants who experience significant VR-related discomfort will discontinue VR sessions and be withdrawn from the study.

Participants will also have access to follow-up support through direct contact with designated research team members via phone or email. Instructions will be provided for reporting any immediate or delayed concerns related to the intervention. A board-certified psychiatrist on the team will be involved in cases of adverse events to help provide referrals and recommend next steps.

### Outcome Measures

Clinical outcome assessments will be collected at baseline, immediately following completion of the intervention, and 6 weeks after the intervention to evaluate the maintenance of any observed effects over time.

Participants will also provide demographic and professional information, including age, gender identity, professional role, clinical setting, primary patient age group, years of professional experience, hours of direct patient care per week, and predominant patient insurance coverage. Participants will also report prior experience with VR and other digital or interactive mental health resources and their tendency to experience motion sickness, dizziness, or nausea.

The primary outcomes of interest are feasibility and acceptability, which will include recruitment, retention, and intervention completion rates. Participant engagement and adherence will be assessed using use data from both intervention formats, including session completion, frequency of use, time spent engaging with intervention materials, and completion of postsession assessments. At the postintervention assessment, acceptability, appropriateness, and feasibility will also be assessed using the Acceptability of Intervention Measure (AIM), Intervention Appropriateness Measure (IAM), and Feasibility of Intervention Measure (FIM) [39], along with participant feedback on satisfaction and overall experience with the intervention.

Exploratory clinical outcomes will include burnout, STS, and self-compassion. Burnout will be measured using the Maslach Burnout Inventory [40]. STS will be assessed using the Secondary Traumatic Stress Scale [41]. Self-compassion will be measured using the 12-item Self-Compassion Scale–Short Form [42].

### Data Analysis Plan

To evaluate feasibility and acceptability, recruitment, retention, intervention completion, adherence, and participant satisfaction will be summarized using descriptive statistics. AIM, IAM, and FIM scores will also be summarized descriptively. These outcomes will be considered alongside the progression criteria described below.

Exploratory clinical outcomes will include self-compassion, burnout, and STS. Changes in these outcomes over time and between study arms will be examined using repeated measures analyses. These analyses will allow us to examine whether outcomes improve over time and whether changes differ between the 2 study arms. Effect estimates and 95% CIs will be reported to inform the design and sample size of a future definitive trial.

Outcomes will be analyzed using a mixed-effects model for repeated measures, with time (baseline, postintervention, and 6-week follow-up) as the within-subjects factor and study arm (VR vs comparison) as the between-subjects factor. The model will include fixed effects for treatment group, time (categorical), and the treatment-by-time interaction. An unstructured covariance matrix will be used to model within-subject correlations across repeated measurements. The between-group mean difference at the final follow-up time point will be estimated from model-based marginal means and reported with 95% CIs. The treatment-by-time interaction will also be examined to determine whether changes over time differ between the 2 groups. Missing outcome data will be handled within the mixed-effects modeling framework, which allows participants with incomplete repeated measurements to contribute available data to the analysis.

Progression to a future definitive trial will be guided by feasibility and acceptability criteria using a proceed, modify, or reconsider framework [43,44]. Recruitment feasibility will be evaluated based on the recruitment rate, the proportion of eligible individuals who enroll, and the ability to recruit participants within the planned study period. Intervention feasibility will be supported if at least 70% of participants complete all 8 intervention sessions. Retention feasibility will be evaluated based on the proportion of participants completing the postintervention and 6-week follow-up assessments. Acceptability, appropriateness, and feasibility will be evaluated using AIM, IAM, and FIM scores and participant feedback, while VR tolerability will be evaluated based on VRSQ responses and reported adverse events. If feasibility concerns are identified but can reasonably be addressed through modifications to recruitment or intervention procedures, progression to a larger trial may occur with modifications. If substantial barriers to recruitment, retention, intervention completion, acceptability, or safety are identified that cannot reasonably be addressed, a definitive trial would not proceed in its current form. Progression criteria will be considered collectively rather than as rigid pass or fail thresholds. Exploratory clinical outcomes will not be used to determine progression [45].

### Ethical Considerations

This study has been approved by the UCSD Institutional Review Board (IRB; 814637), in accordance with the US Department of Health and Human Services regulations for the protection of human subjects (45 CFR 46). All participants will provide informed consent before taking part in any study activities. Participation will be voluntary, and participants may withdraw from the study at any time without penalty.

Research data will be stored on secure, password-protected systems, and access will be limited to authorized members of the research team. Identifiable information will be kept separate from study data whenever possible.

Any changes to the study protocol will be submitted to the IRB for review and approval before implementation. The study was registered on ClinicalTrials.gov prior to participant enrollment.

## Results

The study was funded in April 2026 and subsequently received approval from the UCSD Institutional Review Board and was registered on ClinicalTrials.gov. As of October 2026, 38 participants have provided consent and have been enrolled. Recruitment for a second wave of participants is ongoing, and data collection is currently in progress. Data analysis has not yet begun and is expected to commence in December 2026. Results are expected to be published in spring 2027.

## Discussion

### Anticipated Principal Findings

We expect this pilot study to provide information about the feasibility and acceptability of delivering a brief, self-administered VR-CF-CBT intervention to mental health care professionals. The study will help determine whether mental health care professionals are willing and able to complete brief VR sessions over several weeks, whether the intervention is acceptable and well tolerated, and whether any changes are needed before moving to a larger trial. Recruitment, retention, intervention completion, adherence, acceptability, appropriateness, perceived feasibility, and VR tolerability will be considered together with the prespecified progression criteria to guide decisions about a future trial.

The study will also explore changes in self-compassion, burnout, and STS. Because this pilot is not designed or powered to determine efficacy, these clinical outcomes will be interpreted as preliminary. They will provide estimates of the direction and size of changes and between-group differences that can help with outcome selection, sample size planning, and the design of a future definitive trial.

### Comparison With Prior Work

Previous studies have shown that compassion-based interventions can improve self-compassion and well-being [22,24], while CBT-based approaches can help individuals recognize and change unhelpful patterns of thinking [16-18]. Research has also shown that VR can be an engaging way to deliver mental health interventions and may support emotional regulation, skill development, and compassion-related outcomes [9,13-15,28,29]. However, little research has examined CF-CBT delivered through VR specifically for health care professionals experiencing burnout and STS. This study builds on previous work by applying these approaches to a group that is regularly exposed to stress, trauma, and emotional demands in the workplace. By comparing VR-CF-CBT with an active comparison condition that receives the same core therapeutic content, this pilot may also provide early information on whether immersive delivery is worth further study beyond the content itself.

Evidence on the feasibility and implementation of VR-based interventions among health care professionals is also growing. A 5-week VR relaxation intervention among mental health staff was found to be feasible and acceptable; however, work commitments affected attendance, and the authors highlighted flexible scheduling and self-help delivery as important considerations for future use [46]. A randomized pilot trial of a 1-week home-based VR intervention among health care workers also found that remote VR use was feasible and acceptable, with participants reporting that the system was easy to use at home [47]. Longer approaches have also been tested, including an 8-week VR program targeting burnout and work engagement among health care professionals [48]. At the same time, studies of VR implementation in health care have identified practical barriers such as limited time, limited technical knowledge and support, infrastructure, and access to VR equipment [49,50].

Although these studies support the use of VR among health care professionals, there is still limited evidence on multiweek therapeutic interventions that combine self-guided access with a structured sequence while also measuring feasibility and implementation-related outcomes. The present study explores this approach by providing participants in the VR group with individual access to a headset throughout the intervention period. This allows them to complete sessions at times that fit their schedules instead of being tied to repeated research visits or staff availability. Participants can move through the intervention at their own pace over approximately 4 to 6 weeks, while the treatment sequence remains consistent because each scenario must be completed before the next becomes available. This structured but flexible approach is intended to give participants more control over when they complete the sessions while keeping the intervention content in the same order. Evaluating recruitment, retention, intervention completion, acceptability, appropriateness, perceived feasibility, and VR tolerability within this model may help determine whether a largely self-guided VR intervention can be practically delivered to health care professionals.

### Strengths and Limitations

An important strength of this pilot is the use of an active comparison condition that provides the same core CF-CBT content through digital booklets. This allows the study to compare 2 ways of delivering similar therapeutic content rather than comparing VR with no intervention. The intervention is also brief, self-administered, and designed for mental health care professionals with demanding schedules. Providing participants with individual headset access and flexibility in when they complete sessions may also reduce the need for repeated research visits while still keeping the therapeutic content in a set order. In addition, the study examines several aspects of feasibility, including recruitment, retention, adherence, acceptability, appropriateness, and VR tolerability, along with exploratory clinical outcomes.

Several limitations should also be considered. First, as a pilot feasibility trial with a relatively small sample, the study is not designed to determine whether the intervention is effective. The clinical outcomes are exploratory and will mainly provide information for planning a future trial. Second, because participants will be recruited mainly from health care settings in the San Diego area, the findings may not apply to other health care systems, professional groups, or geographic settings. Third, because the active comparison group receives the same core CF-CBT content, differences between groups may be smaller than in studies that use a minimal-intervention or no-intervention comparison group. Differences in engagement or dropout between the VR and booklet groups could also affect the exploratory comparisons. However, understanding these patterns of engagement and retention is itself an important goal of this feasibility study.

### Future Directions and Dissemination

Findings from this pilot will be used to improve recruitment procedures, intervention delivery, and outcome assessment. The feasibility findings may also help identify whether changes are needed in session timing, participant support, headset distribution, or other parts of the intervention. If the findings support moving forward, a larger and adequately powered study could evaluate clinical effectiveness in a broader range of health care professionals and settings and more closely examine whether immersive VR adds benefit beyond nonimmersive delivery of the same therapeutic content.

Study findings will be shared through peer-reviewed publications, scientific presentations, and relevant institutional and professional networks. Reporting of the completed pilot trial will follow applicable CONSORT guidance for randomized pilot and feasibility trials [31,45]. A summary of study findings will also be made available to participants upon request.

### Conclusions

This pilot will provide important information on whether brief, self-administered VR-CF-CBT can be feasibly and acceptably delivered to mental health care professionals and whether any changes are needed before larger-scale testing. It will also provide preliminary estimates of changes in self-compassion, burnout, and STS to help guide the design of future efficacy trials, but these findings will not be interpreted as definitive evidence of effectiveness. If feasibility and acceptability are supported, the intervention may be suitable for further study across a wider range of health care settings and health care professional groups. A larger multicenter trial could then evaluate its effectiveness and determine whether this approach has value as a scalable support tool for health care systems addressing mental health care professional burnout.

## Appendix

Multimedia Appendix 1 SPIRIT 2025 checklist.

Multimedia Appendix 2 Peer Review Report by the Sanford Institute for Empathy and Compassion, University of California, San Diego (USA).

## Abbreviations

AIM: Acceptability of Intervention Measure
CBT: cognitive behavioral therapy
CF-CBT: compassion-focused cognitive behavioral therapy
CONSORT: Consolidated Standards of Reporting Trials
FIM: Feasibility of Intervention Measure
IAM: Intervention Appropriateness Measure
IRB: institutional review board
SPIRIT: Standard Protocol Items: Recommendations for Interventional Trials
STS: secondary traumatic stress
UCSD: University of California San Diego
VR: virtual reality
VR-CF-CBT: virtual reality–enhanced compassion-focused cognitive behavioral therapy
VRSQ: Virtual Reality Sickness Questionnaire

## Data Availability

The datasets generated and analyzed will be available from the study lead upon reasonable request and in accordance with institutional policies and participant privacy protections.
