# Supplementary material for: Virtual Reality for Enhancing Self-Compassion and Reducing Burnout Among Mental Health Care Professionals: Protocol for a Pilot Feasibility Randomized Controlled Trial
Source: JMIR Res Protoc. 2026 Oct 19;15:e105754. doi: 10.2196/105754 (PMC13728764; doi:10.2196/105754)
Supplement: Supplementary file 2 [file resprot_v15i1e105754_app2.pdf]

UNIVERSITY OF CALIFORNIA SAN DIEGO

BERKELEY • DAVIS • IRVINE • LOS ANGELES • MERCED • RIVERSIDE • SAN DIEGO • SAN FRANCISCO SANTA BARBARA • SANTA CRUZ

SANFORD INSTITUTE FOR EMPATHY AND COMPASSION

9500 GILMAN DRIVE # 0066
LA JOLLA, CALIFORNIA 92093-0602
EMPATHYANDCOMPASSION@UCSD.EDU
URL:EMPATHYANDCOMPASSION.UCSD.EDU

March 2, 2026

Dear Dr. Wael Al-Delaimy,

Congratulations! We are pleased to inform you that your proposal, *Virtual Reality for Enhancing Self Compassion and Reducing Burnout Among Healthcare Providers*, has been selected for **conditional approval upon revision** under the Sanford Institute for Empathy and Compassion. Proposals were ranked on a NIH-review scale of 1-10, and your proposal scored in the top 25% range. Through a competitive review process, this award recognizes the potential of your project. Pending fulfillment of the conditions outlined below, the award will be finalized.

Award Information and Terms:

- **Principal Investigator (PI):** Wael Al-Delaimy
- **Total Award Amount:** *To be determined upon revisions*
- **Purpose:** Virtual Reality for Enhancing Self Compassion and Reducing Burnout Among Healthcare Providers
- **Period:** April 1, 2026 – March 31, 2027

Conditions for Final Approval:

1. Provision of responses and/or revisions to weaknesses by **March 15, 2026**.

<u>*Strengths*</u>

- The proposal is innovative and addresses an important problem, with notable strengths including the use of immersive VR technology, a compassion-focused CBT framework, and strong clinical partnerships in both San Diego and Gaza.
- The authors appropriately acknowledge the limited existing data on the use of this approach in healthcare settings, underscoring the exploratory and pilot nature of the work.

<u>*Weaknessses*</u>

- The overall scope appears ambitious relative to the proposed budget and pilot timeframe. The development, cultural adaptation, and deployment of a VR-based CF-CBT intervention across two highly distinct clinical contexts—a high-resource academic medical center in San Diego and low-resource, post-conflict clinical settings in Gaza—introduces substantial logistical, cultural, and methodological complexity. Given the acknowledged lack of prior data in healthcare contexts, combining these settings within a single pilot may limit feasibility and interpretability, and may be better approached as staged or site-specific studies.
- While the proposal clearly specifies mental health providers at UC San Diego, there is notable heterogeneity within this group in terms of training, clinical roles, and scope of practice (e.g., psychiatrists, psychologists, therapists, and other providers). This variability alone may influence baseline stress, burnout, and responsiveness to the intervention. Beyond UCSD, there is limited detail regarding the types, roles, and practice environments of healthcare providers in Gaza, and the additional heterogeneity across sites further complicates interpretation of outcomes and cross-context comparisons.

- Evaluation plan raises concerns regarding participant burden. The assessment battery includes multiple validated self-report measures (MBI, STSS/ProQOL-STS, SCS, GAD-7, PHQ-9, ERQ), in addition to a cognitive performance task, administered at three time points. While each instrument is individually well-justified, the cumulative burden may be substantial for healthcare providers already experiencing high occupational stress, potentially affecting recruitment, retention, and completeness of follow-up data. This concern is amplified by the absence of participant support or compensation in the proposed budget, which may further challenge enrollment and sustained engagement in a pilot study of this complexity.
- Rationale for key design decisions (e.g., 10 sessions, 4–6 week duration, interval between T1 and T2, and sample size justification) is not clear.
- Analytic plan would be strengthened by specifying procedures to address use of multiple measurements and to control Type I error.
- Absence of a comparison to conventional CBT somewhat limits the ability to determine effect specificity or the unique value of using VR.
- Recommend to not to wait until month 3 or 4 to start recruiting participants. The process is likely going to be long, especially in Gaza.
- Lack of any compensation for participating in the study. While the reward might be "improving self-compassion" in and of itself, participants from Gaza may struggle to find the time to participate.

If you have any questions about your decision letter, please direct them to Cindy Chwa (cchwa@health.ucsd.edu).

Sincerely,

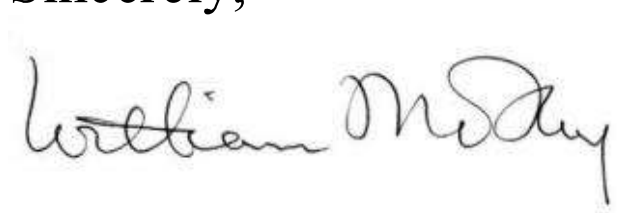

William Mobley, MD, PhD
Professor, Department of Neurosciences
Associate Dean Neurosciences Initiatives
Director, Sanford Institute for Empathy and Compassion
University of California, San Diego School of Medicine
